# Rectal gastrointestinal stromal tumor: an unusual presentation of an uncommon pathology (a case report)

**DOI:** 10.11604/pamj.2021.39.234.26727

**Published:** 2021-08-12

**Authors:** AbdulHafiz Oladapo Adesunkanmi, Olalekan Olasehinde, Chinedu Udochukwu Ndegbu, Tunde Adebowale Odunafolabi, Babatunde Mustapha, Adedayo Olaitan Lawal, Ifeoluwa Seun Olorunsola

**Affiliations:** 1 Department of Surgery, Obafemi Awolowo University Teaching Hospitals Complex, Ile-Ife, Osun State, Nigeria,; 2 Department of Surgery, Obafemi Awolowo University, Ile-Ife, Nigeria,; 3 Department of Surgery, Federal Medical Center Owo, PMB 1053, Ondo State, Nigeria,; 4 Department of Morbid Anatomy and Forensic Medicine, Obafemi Awolowo University Teaching Hospitals Complex, Ile-Ife, Osun State, Nigeria

**Keywords:** Gastrointestinal stromal tumour, rectal GIST, large bowel obstruction, imatinib, case report

## Abstract

Gastrointestinal stromal tumours (GIST) are a rare form of neoplasm. The stomach is the commonest location while gastrointestinal bleeding and pain are the usual presentations. Rectal GIST has been reported in literature as a rare occurrence. We report the rare case of a 37-year-old man who presented with large bowel obstruction and acute urinary retention arising from a rectal GIST. Radiological investigations showed features in keeping with intestinal obstruction. He had a divided colostomy and tumour debulking. Histology of tumour revealed a rectal GIST and immunohistochemical staining was positive for CD34 and CD117. Postoperatively Imatinib was commenced and patient did well. We report this case to highlight the unusual symptoms that may arise from a rare pathology like rectal GIST and the need to consider an alternative diagnosis-such as GIST, in a young adult presenting with large bowel obstruction in the absence of risk factors for bowel adenocarcinoma.

## Introduction

Gastrointestinal stromal tumors are neoplasms of mesenchymal origin believed to arise from gut pacemaker cells known as “interstitial cells of Cajal” [1]. They account for 0.3-1% of all primary gastrointestinal tumors [2]. Despite the relatively low incidence of GIST compared to primary gastrointestinal adenocarcinomas, it remains an important differential of gastrointestinal tumors because of its location, pattern of presentation, and potential for cure owing to the availability of immunohistochemical typing and targeted therapy. Rectal GIST accounts for 5% of all GIST occurring either alone or extending to the anal, prostate, or urinary bladder [3]. Such rare presentations have often constituted clinical diagnostic dilemma especially if presenting initially in the acute setting.

The pattern of presentation of GIST varies widely. Although some cases may be asymptomatic, usual symptoms include gastrointestinal bleeding, unexplained anaemia, palpable abdominal mass, or abdominal pain from perforation or pressure effect [4]. These symptoms can easily be mistaken for primary gastrointestinal adenocarcinoma. Few reports of rectal GIST exist in literature and cases described highlight rectal bleeding as the usual presentation. The occurrence of large bowel obstruction from rectal GIST is a far more unusual scenario, which becomes more intriguing when associated with urinary symptoms. This case report highlights presentation of GIST in a 37-year-old gentleman which is considered atypical by virtue of its unusual rectal location and consequent large bowel obstruction.

## Patient and observation

**Patient information:** a 37-year-old man presented to the acute surgical care unit of our institution with a 2-day history of progressive abdominal distension, colicky lower abdominal pain, and an episode of faeco-bilious vomiting. There was a preceding history of gradual change in bowel habit over a three-month period characterized by episodes of constipation alternating with diarrhea, passage of mucoid stool and tenesmus but no history of hematochezia. In addition, he had an episode of acute urinary retention a day before presentation which required urethral catheterization at a peripheral clinic. Additional symptoms include pain on the inner aspect of his right thigh and a recent history of erectile dysfunction. No significant history of preceding lower urinary tract symptoms. There is no family history of a colon or rectal malignancy known to the patient. Throughout the period of his symptoms, he noticed significant progressive weight loss.

**Clinical findings:** at presentation to the acute surgical care unit, he was dehydrated, tachycardic but apyrexic. Key findings were on abdominal examination which revealed grossly distended abdomen that was tense with vague generalized tenderness but no palpable umbilical nodule or abdominal mass. Percussion note was tympanic and bowel sounds were hyperactive. Rectal examination revealed firm non-tender extraluminal mass about 5cm from the anal verge, which was bulging into the rectal lumen from the anterior rectal wall and completely occluding the rectal antrum. The distal lumen was empty and the overlying mucosa was intact. Gloved examining finger was smeared with clear mucus.

**Figure 1:**
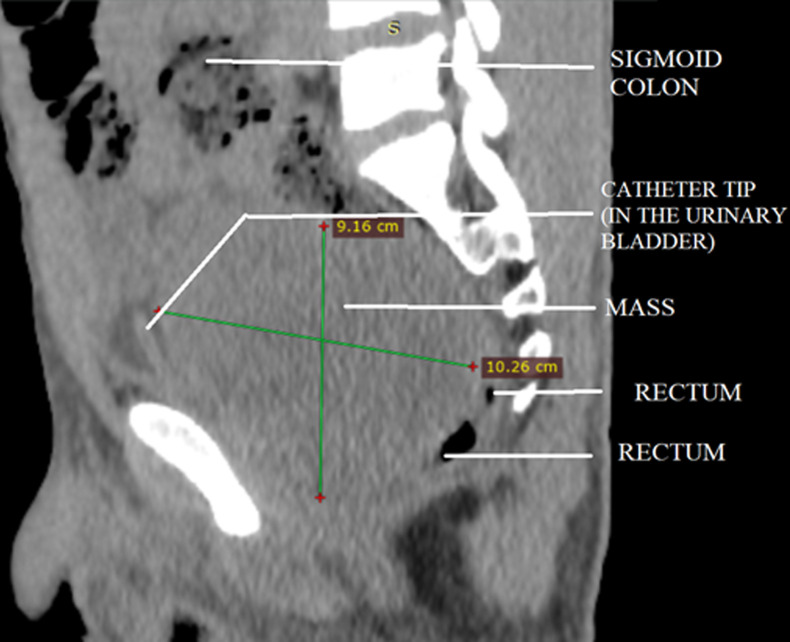
sagittal view of non-contrast abdominopelvic computerized tomography scan showing huge pelvic mass indistinguishable from the anterior rectal wall, with an anteriorly displaced urinary bladder containing a catheter

**Figure 2:**
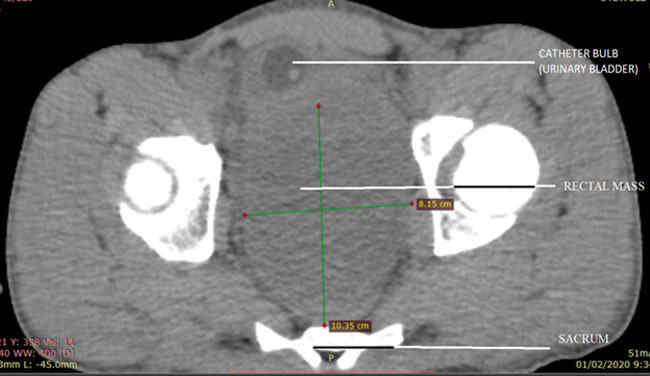
axial view of the pelvis at the level of the hip joint further demonstrating the pelvic mass, inseparable from the rectum and completely obliterating its lumen while displacing the urinary bladder anterolaterally

**Diagnostic assessment:** plain abdominopelvic radiograph revealed dilatated bowel loops with multiple air-fluid levels having both small and large bowel signatures. Full blood count and electrolytes were unremarkable except for mild azotaemia. Non-contrast abdominopelvic computerized tomography (CT) scan was performed which confirmed a pelvic mass indistinguishable from the anterior rectal wall, with pressure effect on the bladder and prostate gland anteriorly (Figure 1, Figure 2). Fat plane between the lesion, prostate gland, and bladder was however preserved. Prostate scan and serum prostate-specific antigen assay were essentially normal.

**Diagnosis:** a presumptive diagnosis of large bowel obstruction from an advanced pelvic mass was made. Following these findings, he was resuscitated using nasogastric decompression, intravenous fluids, and empirical intravenous antibiotics.

**Therapeutic intervention:** he underwent emergency laparotomy, tumor biopsy, and divided sigmoid colostomy. Intra-operative findings were grossly dilated small and proximal large bowel loops extending up to the proximal third of the rectum and 12 x 8 x 5cm firm mass arising from the anterior rectal wall with central area of necrosis. The mass was seen bulging into the rectum posteriorly while occupying the entire recto-vesical pouch and displacing the urinary bladder anteriorly. No evidence of faecal or urinary spillage was encountered. Debulking tumor resection was undertaken to remove the necrotic central tumor area, while viable areas of the tumor were biopsied. The integrity of the rectal mucosa was confirmed after necrosectomy and biopsy. No liver or gross peritoneal metastasis was seen. There were no palpable pelvic lymph nodes encountered.

**Figure 3:**
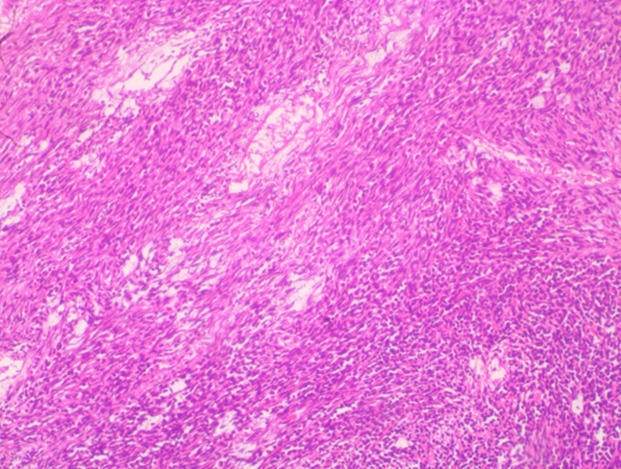
photomicrograph showing proliferating spindle and oval-shaped cells that are disposed in sheets and fascicles; they have elongated, wavy, hyperchromatic nuclei and scanty eosinophilic cytoplasm (H & E x 100)

**Figure 4:**
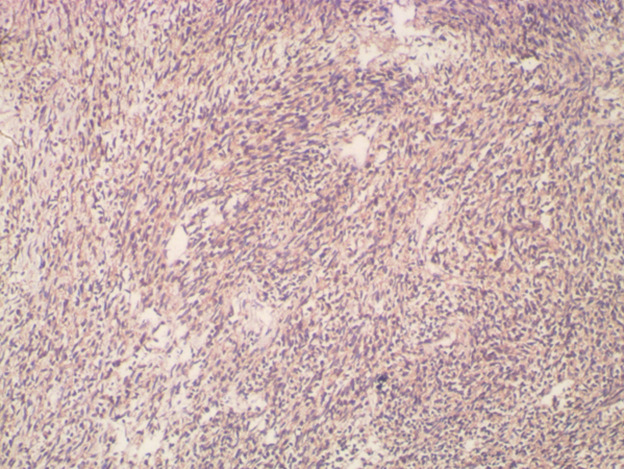
photomicrograph showing strong and diffuse cytoplasmic staining for CD117 (DAB x 100)

**Follow-up and outcome of interventions:** post-operatively, stoma started functioning on the 3^rd^ postoperative day and oral intake was commenced the same day. He was discharged on the 13^th^ day after surgery. Histopathologic examination of biopsy specimen reported malignant gastrointestinal stromal tumour. Immunohistochemical staining was positive for CD34, CD117(C-KIT), and DOG-1 (Figure 3, Figure 4). Imatinib was commenced on the 2nd week after surgery. After 4 weeks on targeted therapy, he has gained weight (from 59kg at discharge to 62kg at the last clinic visit) and has no clinical features of disease progression. Repeat abdominopelvic CT scan scheduled to assess tumor response to Imatinib could not be performed due to financial constraints.

**Patient perspective:** before I came to the hospital I was in a bad shape. After I was treated and had the operation there has been a remarkable improvement. I feel comfortable using the imatinib and I have gained weight. I still feel a little bit uncomfortable with the colostomy but I am getting used to it.

**Informed consent:** the patient gave informed consent to have his case reported and published after he had been counselled about his case, the reason why his case is unique, and the invaluable benefits of documenting the findings.

## Discussion

The index case details an uncommon presentation of large bowel obstruction and acute urinary retention resulting from rectal gastrointestinal stromal tumour in a young patient. The case, with its full immunohistochemical characterization, to the best of the author's knowledge, is among the few cases of rectal GIST reported so far in sub-Saharan Africa [5]. It highlights not only the rarity of rectal GIST and the possibility of atypical symptoms but also emphasizes the need to consider unusual aetiology in young adult patients presenting with symptomatic rectal mass.

Gastrointestinal stromal tumour (GIST) are rare tumours of the gastrointestinal tract, despite being the most common mesenchymal tumours of the gastrointestinal tract [6]. The overall incidence of GIST is estimated as 10-20 per 100,000 population including incidental tumors [1]. The peak age for presentation is reported to be between 50-70 years with equal gender distribution [7]. The occurrence of this tumor before the 5^th^ decade is uncommon. Although GIST can appear anywhere within the gastrointestinal tract, the stomach remains the most frequent location accounting for up to 60% of cases [3]. Other possible sites include small intestine (25%), rectum (5%) and colon (1%) [3]. The index patient is in his 4^th^ decade and falls outside the peak age widely reported in literature. He also has a rectal GIST which is classed as one of the rare locations and is reflected in the paucity of such cases available in the literature. All these further highlight the peculiarity of his presentation.

GISTs vary considerably in their presentation and clinical course. Some reported presentations of rectal GIST from literatures reviewed include anal pain, constipation and haematochezia [5]. Our patient presented in acute intestinal obstruction with an episode of acute urinary retention. The history of right lower limb pain, as well as erectile dysfunction, are rarely mentioned in literature. These features can be attributed to the mass effect of the tumour by virtue of its size. The fact that these symptoms improved remarkably following surgery and the commencement of targeted therapy lays credence to this plausible explanation. Initial evaluation and assessment of the extent of GIST require imaging modalities which provide a reasonable level of information to support a diagnosis of GIST. Modalities often deployed include a contrast-enhanced computed tomography scan (CT scan) which is the standard method of GIST imaging [8]. Magnetic resonance imaging (MRI) is especially useful in patients in whom CT scan is contraindicated. It is also useful for liver-specific lesions. Fluorodeoxyglucose positron emission tomography (FDG-PET) also has good specificity and sensitivity for evaluation of tumor response after Imatinib treatment [3]. In resource-constrained settings like ours, these radiological investigations are not routinely available in many hospitals, and even when available they are quite expensive. The reliance on clinical acumen and histopathological diagnosis of resected specimen are therefore not uncommon in our clime.

Definitive diagnosis of GIST is based on a combination of tissue morphology and immunohistochemical staining criteria [9]. Gastrointestinal stromal tumours (GIST) are typically immunoreactive for KIT. KIT (CD117) is a transmembrane receptor that is part of the tyrosine kinase receptor complex. CD117 positivity is seen in about 90-100% of GIST while positivity for CD34, the hematopoietic progenitor cell antigen, is reported in 70-80% of GIST [10]. The standard treatment of localized GIST is surgical resection with curative intent. About 30 to 50 percent are malignant thus it's advised that all lesions be resected [10]. The most significant factor related to outcome is complete resection of this tumour, and this can be accomplished in 40-60% of all GIST patients. Rectal GISTs have a high rate of local recurrence regardless of the surgical procedure. Depending on the extent of the tumour, local resection, low anterior resection, abdominoperineal resection (APR), and pelvic exenteration are surgeries that can be performed. In each procedure, the aim is complete gross resection with negative microscopic margins [3]. In advanced cases, surgical resection combined with adjuvant Imatinib is expected to improve surgical outcomes and survival. The evidence from randomized trials supports 36 months of adjuvant Imatinib in high-risk GIST [7].

Indices for prognostication include the size, mitotic index, location of the tumour, intratumoral necrosis, aneuploidy, metastatic disease, and the age of the patient [1]. It is also important to note that Gastric GISTs have a better prognosis compared to small bowel and rectal GIST tumors. This index patient had multiple poor prognostic factors (tumor size, rectal location, incomplete excision) and for these reasons was commenced on Imatinib therapy. Unfortunately, paucity of funds and absence of health insurance has hindered adequate monitoring of his oncologic response afterward.

## Conclusion

This case report highlights the need to consider the possibility of rare lesions such as GIST when evaluating a rectal mass in young adult patients having no identifiable risk factors for rectal adenocarcinoma. It also emphasizes the possibilities of unusual symptoms that may be associated with a rectal GIST.
